# Sustaining Integrated Care for Vulnerable Youth: A Longitudinal Case Study of the Regina Intersectoral Partnership (TRiP)

**DOI:** 10.5334/ijic.10258

**Published:** 2026-10-28

**Authors:** Akram Mahani, Adhika Ezra

**Affiliations:** 1 Johnson Shoyama Graduate School of Public Policy, University of Regina, CA; 2 Department of Sociology and Social Studies, University of Regina, CA

**Keywords:** cross-sectoral collaboration, TRiP, vulnerability, youth mental health, integrated care, Tuckman’s group development

## Abstract

**Introduction:**

Many children and youth with mental health challenges face fragmented service delivery and poor cross-sector coordination. This paper examines the Regina intersectoral Partnership (TRiP), a collaborative initiative addressing youth vulnerabilities in Saskatchewan, Canada. The study examines how collaboration within TRiP evolved, adapted, and was sustained since its launch in 2010, situating this evolution within Tuckman’s group development model.

**Methods:**

We employed a qualitative, multi-method design, including 25 semi-structured interviews with policymakers and service providers from six TRiP partner organizations, document reviews, and observations of TRiP meetings. Data were analyzed using reflexive thematic analysis in NVivo 12.

**Results:**

TRiP’s evolution unfolded across four interconnected phases: partnership formation, adaptive implementation, evaluation-driven refinement, and advanced integration. Key enablers—including champions, shared vision, co-location, structured processes, and ongoing evaluation—supported stronger coordination, relational trust, continuous adaptation, and resilience as the initiative matured.

**Conclusion:**

TRiP demonstrates that sustaining integrated care over time requires adaptive governance, organizational learning, strong relationships, and clear collaborative structures. Applying Tuckman’s model shows that long-term cross-sector collaboration is not a fixed progression through stages, but an iterative process in which forming, storming, norming, and performing can overlap and recur as partnerships adapt to changing needs and system conditions.

## Introduction

Many children and youth with mental health conditions are unable to access adequate support in a timely manner [1]. This challenge is largely due to fragmented cross-sectoral services and limited coordination among care providers [2]. In Saskatchewan, a 2019 survey of more than 10,500 Grade 7–12 students found that 23% of students had suicidal thoughts, 21% engaged in self-harm, and over 38% experienced symptoms of depression [3]. While the integration of health and social care systems is inherently complex [4], weak cross-sectoral coordination reinforces silos across health, justice, and other social sectors and human service organizations, resulting in service gaps and inefficiencies [5]. These gaps and shortcomings disproportionately affect vulnerable children and youth, who face the greatest barriers to accessing continuous and person (people/family)-centred care [6].

Cross-sectoral collaboration–the process of aligning organizational resources and capabilities across sectors to address issues that exceed the capacity of any single sector [7]– has been proven to enhance service coordination and improve health and safety outcomes for children [8]. Mental health challenges, in particular, are more effectively addressed through integrated care approaches that enhance service access and reduce fragmentation [9]. Such approaches are most effective when developed and implemented systematically yet flexibly, tailored to children and family needs, and supported by the active participation of families [10]. However, efforts to integrate care services are met with various challenges, including time constraints, unclear roles and responsibilities, the inherent complexity of the problems being addressed, resistance to collaboration, and competing sectoral priorities [10,11].

This paper builds on the work of Honisett et al., [12] which demonstrated the effectiveness of integrated hubs in improving children’s mental health, by examining a case study of cross-sectoral collaboration addressing youth vulnerabilities in Regina, Saskatchewan. It addresses a gap in understanding how integrated youth service partnerships evolve, become institutionalized, and sustain collaboration over time in responding to complex youth needs [13]. Specifically, we examine factors influencing The Regina intersectoral Partnership (TRiP), a longstanding cross-sectoral initiative involving health, education, justice, policing, and social service organizations supporting vulnerable children and youth in Saskatchewan. TRiP represents a valuable case for examining integrated care because of its sustained cross-sector collaboration, its evolution across multiple phases of development, and its emphasis on coordinated family-centered responses to complex youth vulnerabilities. Using a qualitative multi-method design and applying Tuckman’s stages of group development [14], this study provides insights into the development and sustainability of cross-sectoral collaboraiton over time. The lessons derived from this case study offer valuable insights for future integrated care initiatives by highlighting both the conditions that support success while also identifying potential barriers.

We contextualize TRiP’s process using Tuckman’s stages of group development (forming, storming, norming, performing) [14]. The model is widely regarded as a foundational framework to explain the evolution of group dynamics and collaborative functioning (see Bonebright, 2010) [15], but contemporary studies show it is not always a clean, linear progression. For example, recent empirical research suggests that groups may revisit earlier stages, experience overlapping stages, or shift back and forth depending on context and group composition (e.g., Zulkifli et al., 2025) [16]. By applying this model, we aim to better understand how TRiP moved through, and continues to navigate, the complexities of cross-sector collaboration.

## Background and Context

TRiP is a collaborative initiative in Regina, Saskatchewan, that brings together six human service organizations: the Ministry of Social Services; Ministry of Corrections, Policing & Public Safety; Saskatchewan Health Authority; Regina Public and Catholic School Divisions; and the Regina Police Service. The initiative seeks to enhance coordination across sectors to reduce vulnerabilities among children and youth at risk of mental health disorders, crime, violence, school absenteeism, disruptive behaviour, and substance use. In the context of TRiP, vulnerability refers to an elevated risk of adverse outcomes across multiple domains, including mental health challenges, educational disruptions, and limited access to supportive social networks [17]. Recognizing that these complex challenges are often intensified by fragmented service delivery and institutional silos, TRiP was established to improve coordination across sectors and enhance the safety and well-being of vulnerable children and youth. Following an initial period of research on promising practices in collaborative service delivery, the initiative launched its first program in 2010. TRiP delivers long-term case management and tailored service referrals, functioning as a single point of contact that integrates otherwise fragmented institutional and community-based services [18].

Fourteen workers from six partner organizations constitute the core implementation team or the backbone structure of the initiative. Each member holds a defined role: coordinator (n = 1), initiative strategist (n = 1), administrative support (n = 1), intake and referral officer (n = 1), school engagement worker (n = 1), public school liaisons (n = 2), Catholic school liaison (n = 1), sector representatives from justice, social services, and health (n = 3), Jordan’s Principle coordinator for the Indigenous community (n = 1), and support workers (n = 2).

Through its dedicated administrative structure, and with the consent of caregivers or legal guardians, TRiP oversees cases from intake through to closure, with exit determined by a jointly agreed-upon level of readiness to transition out of the initiative. The intake process begins with an interview involving caregivers, the child, and schools, conducted by the Intake and Referral Officer to assess the child’s needs. Following intake, the intersectoral Collaboration Team (iCT) reviews the case and decides on acceptance to the initiative.

For accepted cases, TRiP applies an integrated case management approach to identify the tailored resources required to support each child. This is followed by a Coordinated Custom Case Conference (C4), organized with the caregiver, to develop a tailored action plan responsive to the needs of the child and family. Implementation of the action plan is designed to reduce vulnerability and enhance stability. When both the TRiP team and caregivers agree that sufficient progress has been achieved, a transition plan is developed to support case closure [19]. On average, TRiP’s involvement spans 19 months for children under the age of 11, and 14 months for those aged 12 and older [19].

Since its launch in 2010, TRiP has received over 900 referrals across its 11 and Under Initiative (11UI) and twelve&up programs [20]. These programs provide coordinated, cross-sectoral supports for children and youth at risk of criminal involvement, victimization, school disengagement, mental health challenges, and other forms of vulnerability.

The most frequently identified needs among children and youth referred to TRiP include mental health support and counseling [21]. Independent evaluations have reported positive outcomes, with 94% of children and youth maintaining or improving their school attendance after receiving coordinated support services [21]. Participants also reported personal growth, attributed to a safe learning environment facilitated by TRiP through access to pro-social activities and support workers who served as positive role models. Caregivers emphasized the dependability of services, particularly accessible case managers and trust-based relationships, which supported stronger caregiver–child relationships and improved system navigation [22].

TRiP exemplifies integrated care through both whole-of-system and process-based perspectives. Aligned with Leutz’s (1999) [23] systemic approach, TRiP integrates multiple human service systems with the shared aim of improving outcomes for vulnerable children and youth. It also reflects Kodner and Spreeuwenberg’s (2002) [24] process-based approach to integrated care by coordinating administrative, organizational, and service delivery mechanisms to enhance cross-sector communication, streamline referrals, and align community and institutional resources. In doing so, it functions as an integrated, cross-sectoral model of prevention and early intervention. Using Tuckman’s stages of group development, this paper examines TRiP’s evolution over time and identifies key factors that enabled and constrained collaboration within a long-standing cross-sectoral partnership.

## Methods

### Study Design

This research employed an interpretivist qualitative single-case study design to examine the evolution of TRiP’s collaborative initiative, from its formation through implementation to ongoing sustainability. A qualitative multi-method approach was adopted to enable a richer understanding of collaborative processes and to strengthen triangulation across data sources [25]. Data collection included semi-structured interviews, document reviews, and direct (non-participant) observations of meetings. This paper reports findings from a broader study of cross-sectoral collaboration for children and youth in vulnerable contexts. Ethics approval for the study was granted by the University of Regina Research Ethics Board (REB-2023-169).

### Data Collection

**Interviews:** A total of 25 semi-structured interviews were conducted between May 25 and August 31, 2023 by the project lead (first author). Recruitment started in early April 2023 using purposive, snowball, and convenience sampling strategies to recruit participants with knowledge and experience of collaborative processes within TRiP [26]. Participants included policymakers and service providers representing six partner organizations actively engaged with TRiP. The sample consisted of current and former members of the TRiP Steering Committee (n = 11), past and present frontline staff or backbone team (n = 9), and senior policymakers and policy entrepreneurs (or champions) with extensive familiarity with TRiP (n = 5), including two who played key leadership roles in the initiative’s early development. This ensured representation from all human service sectors.

Interviews were conducted via Zoom, with each session lasting a minimum of 60 minutes. Written informed consent was obtained prior to participation. All interviews were audio-recorded with participants’ consent, and transcribed verbatim using Otter, an automated voice-to-text transcription software. Two trained undergraduate students conducted quality checks on all transcripts to ensure that Otter accurately captured the interviewees’ responses.

**Document Review:** To contextualize and corroborate the interview findings, an extensive review of TRiP-related documents was undertaken. These included internal and external evaluation reports, intake and referral forms, needs assessment tools, steering committee process documents, and relevant webpages hosted on the TRiP website. Documents were accessed with organizational permission under the approved ethics protocol and did not include identifiable client or family information.

**Observations:** To further corroborate and enrich interview findings, the project lead conducted non-participant observations of four TRiP meetings between November 2023 and January 2024: an intersectoral Collaboration Team (iCT) meeting, a case conference, a TRiP team meeting, and a steering committee meeting. Observational methods are commonly used in qualitative case study research to capture relational dynamics, interactions, and organizational processes in naturalistic settings [27]. As coordinator of the broader research project, the project lead had established rapport and trust with the TRiP team, facilitating access while minimizing disruption to routine interactions. Observations focused on collective decision-making, coordination of services, communication processes, power dynamics, accountability mechanisms, and management practices. Field notes were taken during each observation; no audio recordings were made.

### Data Analysis

Data from interviews, documents, and observations were analyzed using reflexive thematic analysis [28]. The process followed six iterative phases: (1) data familiarization, (2) generating initial codes, (3) reviewing and refining codes, (4) identifying patterns and themes across datasets, (5) developing categories and subcategories, and (6) finalizing and defining themes. A hybrid deductive–inductive coding approach was employed. Initial deductive codes were informed by the interview guide and scholarship on cross-sectoral collaboration, while inductive coding allowed additional patterns and insights grounded in the data to be incorporated throughout the analytic process. Two graduate students coded transcripts, documents, and observational notes in NVivo 12 under the supervision of the project lead. Regular analytic meetings were held to refine coding decisions and interpretations across datasets. Analytic memoing [29] was used throughout the process to document reflexive insights, coding decisions, and relationships across datasets. Emerging interpretations and thematic categories were also discussed with the broader research team through peer debriefing to strengthen analytical rigor and reflexivity [30]. Triangulation across interviews, document reviews, and observations was used to compare findings across data sources, corroborate interpretations, and enhance contextual depth and credibility. This approach enabled a more comprehensive understanding of collaborative processes, organizational dynamics, and the evolution of TRiP over time [31].

## Results

Since its launch in 2010, TRiP has evolved through changing community needs, organizational learning, and ongoing adaptation. To capture the longitudinal nature of the initiative, the findings are organized into four interconnected developmental phases that broadly align with Tuckman’s stages of group development. While presented sequentially, these phases often overlapped as TRiP evolved in response to emerging challenges and service gaps. Phase 1 focuses on the early formation of the partnership, including shared problem recognition, the establishment of common goals, the role of champions, and the creation of foundational governance structures. Phase 2 marks the transition from planning to implementation, highlighting program expansion, operational refinement, co-location, and the strengthening of collaborative processes. Phase 3 examines how formal evaluation, organizational learning, and system refinement contributed to deeper integration while also exposing ongoing structural and operational challenges. Finally, Phase 4 highlights TRiP’s continued maturation through broader system integration, innovation, resilience, and sustained adaptation in response to evolving community and organizational needs. Across all phases, TRiP’s evolution was shaped by champions, relational trust, and adaptive collaboration in response to systemic gaps and complex social needs.

### Phase 1: Early Formation and Shared Problem Recognition (2008–2010)

#### Shared Problem Identification and Definition

The absence of proactive, preventive mechanisms to keep youth from entering the criminal justice system was first recognized in 2008 by the Regina Police Service (RPS) and the Regina Public School Division. This shared recognition laid the foundation for cross-sectoral collaboration. Representatives from the RPS realized how policing alone could not address youth vulnerability within a fragmented and reactive system. One participant emphasized the limitation of policing–restricted to work within the legal system–to address a complex social issue. Speaking on the criminalization of youth delinquency arising from unstable living conditions and inadequate social support, the participant reflected how it “*wasn’t necessarily a very productive way to manage the problem… we needed an entirely different approach to what we are doing if we are ever going to break the cycle*” [P5].

Other sectors similarly recognized the limitations of working in isolation, highlighting the need for cross-sectoral coordination. As one participant explained:

“Historically, the government has been set up within service lines; ministries fall within service lines. And that’s useful for organizational or budget purposes, but it is not useful from a client perspective, because somebody who needs a collection of services…doesn’t want to go to multiple places or partners to access them. So, we in government recognize that our structure is maybe not well-suited to meeting public needs.” [P3]

From this recognition, RPS and Regina Public Schools, who likewise acknowledged the limitations of the education system in addressing broader social issues facing children and youth, entered into dialogue. This process led to the articulation of a shared goal that became the ideological foundation of the TRiP initiative. One participant emphasized the importance of creating an initiative able to “*blend all of the strands that seem to float in the air within our community, that are either trying to work towards similar kinds of outcomes, or are fighting to have their voices heard*” [P23]. Through this process, the initiative’s focus broadened from reducing youth contact with the justice system to the shared goal of “*universally improving the safety and well-being of children and youth*.”

#### Champions as Catalysts for Partnership Formation

During the partnership development stage, champions, who were motivated leaders willing to challenge institutional norms and advocate for cross-sectoral solutions, played a critical role in building credibility, mobilizing partners, and sustaining momentum. Their ability to navigate conflicts and engage in difficult conversations constructively was viewed as essential for fostering open dialogue. As one participant noted, the champion could “*have uncomfortable and difficult conversations, but in a way where it was safe and open*” [P13].

Champions also brought an understanding of the strengths and operational realities of different sectors, including resource and budget considerations, which supported effective coordination and resource allocation. Combined with their direct engagement with frontline staff and families, this cross-sectoral understanding positioned champions as the “*glue that holds everything together*” [P17].

Their ability to communicate across different audiences was equally important for building trust and support for TRiP. Champions adapted their language and messaging to engage diverse stakeholders, from Steering Committee members to families and community partners. As one participant explained, “*the conversation they would have with the Steering Committee when they present their report would be very different to when they’re in the community speaking with a family… or whether they’re in the community trying to garner support through sponsorship*” [P17].

#### Building a Shared Vision and Priorities

All partners coalesced around a shared commitment and vision: to improve outcomes for vulnerable children, youth, and their families. This shared vision created early buy-in and provided a solid foundation for collaboration. As the initiative progressed, its ability to adapt to the complexity of youth vulnerability allowed it to better respond to community needs. Efforts increasingly focused on integrating family needs, addressing mental health, and adopting a more holistic, coordinated approach to supporting youth well-being.

Participants highlighted that this collective commitment, combined with TRiP’s adaptive capacity, strengthened cohesion and sustained momentum over time. One participant described the importance of recognizing a common purpose across agencies:

“I always like to ask, ‘What is our goal?”…’ Not ‘what is MSS [Ministry of Social Services] goal?’… But what is our goal for this youth?… If we can kind of get people to understand that our goal is the same…We can find ways to work together to achieve that within our own agencies.” [P24]

However, emerging challenges began to surface even in this early phase. Limited personnel and funding restricted the ability of partner organizations to engage fully, and some agencies were initially hesitant to commit resources due to competing priorities. This created early constraints in planning and required champions to actively negotiate buy-in from reluctant partners. Early recognition of institutional silos also surfaced, as participants described how mandates and professional boundaries limited cross-sector collaboration even at this formative stage:

“If you work in health or social services, a lot of things are kind of seen as health or social services specific, and you don’t get as much work or agreement on certain things…those would be the biggest reasons as to why that siloing tends to happen.” [P12]

Over time, partners increasingly acknowledged that a coordinated, cross-sectoral approach was essential, reinforcing the shared priorities that guided the initiative forward.

#### Establishing Governance and the Steering Committee

In this early phase, TRiP staff conducted foundational research, explored comparable initiatives, and adapted promising practices to the Regina context. After approximately 18 months of research and development, the first TRiP Steering Committee was formally established in 2010, marking the transition from conceptual planning to structured governance. The committee included the Regina Police Service, Saskatchewan Health Authority, Regina Public Schools, Regina Catholic Schools and the Ministry of Social Services. The Ministry of Justice joined later in 2015 as TRiP’s scope expanded.

### Phase 2: Implementation, Structural Consolidation, and Adaptive Expansion (2010–2015)

Following the initial planning period, TRiP entered a phase of active implementation and structural consolidation. The initiative began with a Steering Committee, Referral Officers, and a Working Group (backbone team or frontline workers). As responsibilities increased, the initiative added a coordinator role in 2011 to oversee day-to-day implementation, supervise frontline workers, maintain partner relationships, prepare reports, and act as the key liaison between the Steering Committee and the Working Group [32]. In the same year, the provincial government released “Building Partnerships to Reduce Crime”, a report that explicitly aligned with TRiP’s approach and provided the initiative with greater legitimacy and political support.

#### Implementation and Strengthening Governance

The first program, 11UI, launched in 2010, marking TRiP’s transition from planning to action. Partners soon identified the need for structured evaluation to assess whether the initiative was producing the intended outcomes. In 2012–2013, with new funding from the Ministry of Social Services, formal quarterly and annual reporting processes were introduced [33]. In 2012, an independent consultant collaborated with the Steering Committee to design indicators that could meaningfully capture behavioral change among children and youth engaged with TRiP.

From July 2013 to June 2014, TRiP completed its first formal evaluation. Results showed a significant reduction in police interactions (from 4.1 to 0.5 contacts on average), along with lower assessed risk of antisocial behavior [34]. These early findings demonstrated positive outcomes while also identifying gaps in the reporting system, leading to further refinement of monitoring processes and, subsequently, a more comprehensive evaluation.

#### Program Adaptation and Expansion

As implementation progressed, TRiP partners recognized a key limitation: children often stabilized until age 12 but required ongoing support into adolescence. The initial mandate to exit clients at age 12 hindered continuity of care. To address this, twelve&up program was launched in 2014 as an extension of 11UI for youth aged 12–18. While grounded in the same principles as 11UI, twelve&up added a deliberate focus on preventing recidivism and strengthening family-level supports, acknowledging that parent and caregiver engagement is crucial to sustained stability. Participants viewed ongoing adaptation as central to TRiP’s longevity:

“It is in need of more evolution today. So it’s an ever-growing, ever-changing sort of initiative… If you settle for the status quo, you will disappear.” [P20]

This adaptive culture contributed to sustained relevance, enabling TRiP to address changing community needs and avoid complacency.

#### Strengthening Collaboration Through Co-Location

Another key development in this phase was the recognition that deeper cross-sectoral coordination required shared physical space. In 2015, TRiP secured an office within the Regina Public School Division Board’s building, and staff moved in later that year [33]. Co-locating partners improved the timeliness and quality of communication, facilitated real-time problem-solving, and strengthened relational trust. As one participant noted: “*TRiP brings together all the agencies at one time, which allows us to focus on one meeting rather than potentially booking five to six different meetings*” [P20]. Co-location also enhanced the ability to monitor clients and share information efficiently [21].

#### Strengthening Operational Tools and Processes

During this period, TRiP refined its operational infrastructure to better support frontline work. Updated referral forms, iCT planning sheets, vulnerability scoring tools, and other reporting mechanisms were introduced to ensure more consistent risk detection and timely intake processes. These tools helped enhance service quality and standardize workflows across sectors. TRiP also developed a Functioning Process Document to clarify partner roles and interactions within the multi-sectoral network, supporting consistency and shared understanding [21].

### Phase 3: Evaluation-Driven Refinement and Institutional Deepening (2015–2018)

Phase 3 marked a pivotal period in which evaluation became both a developmental tool and a catalyst for institutionalization. Building on the early implementation structures established in Phase 2, partners sought a deeper understanding of TRiP’s effectiveness and sustainability. Evaluation was not viewed as an external requirement but as an essential mechanism for legitimizing the initiative, ensuring accountability, and maintaining partner commitment. As one participant emphasized:

“*Because that constant demonstration and reminder of value to everyone is really important to ensure that people are going to continue to buy in and invest into a program. If they feel it’s just a lost ship with little value, it will be gone. They just won’t commit to it.*” [P5]

#### Evaluation as a Driver of Learning and Legitimacy

Beginning in summer 2015, the Living Skies Centre for Social Inquiry initiated a comprehensive, mixed-method evaluation of both 11UI and twelve&up programs. The evaluation framework and instruments were further refined that fall, and extensive data using interviews, surveys, and observational assessments were collected between October 2015 and May 2017 [21].

At the same time, TRiP staff engaged in a parallel internal planning process aimed at strengthening the initiative’s overall measurement capacity. These internal discussions clarified the purpose of evaluation, identified the types of data needed to assess client outcomes, and informed the development of tools to better capture organizational performance. This process strengthened TRiP’s reporting and measurement systems, enabling more systematic assessment of client needs and monitoring of program progress over time [33].

Findings from the 2017 evaluation indicated positive outcomes across several measures [21]. Among clients for whom data were available, 65.9% showed a reduction in vulnerability. School engagement also improved, with 94.3% of students maintaining or increasing their attendance and 86.2% demonstrating “beginning” or “good” academic performance. Family feedback was similarly positive: 100% of children surveyed reported receiving helpful support, 90% of caregivers perceived positive changes in their child, and 82% reported feeling personally supported.

#### Adaptations in Response to Evaluation Findings

Evaluation findings directly prompted several modifications to the initiative aimed at addressing persistent gaps. One early issue that was highlighted in both annual reporting and partner observations involved chronic absenteeism, which was strongly associated with youth vulnerability. In response, TRiP introduced a two-year Student Engagement Worker (SEW) pilot (September 2015–August 2017) to support youth with significant school disengagement and justice system involvement. The SEW became an essential bridge between TRiP and school-based supports, engaging with each student an average of five times [21]. This targeted role aligned the initiative more closely with provincial education priorities and strengthened TRiP’s credibility within the school system.

During this same period, TRiP expanded the partnership table: the Ministry of Justice formally joined in 2015, reflecting both the program’s growing complexity and its increasing institutional recognition.

#### Strengthening Administrative Infrastructure and Data Systems

A major outcome of Phase 3 was the development of more sophisticated administrative tools that supported risk identification, coordinated case planning, and consistent data collection. These included the creation of the iCT Action Plan, the development of a Risk-Based Needs Assessment tool that consolidated multiple data sources into a scoring system, and a standardized process for triaging high-risk clients (scores above 19) through Custom Coordinated Case Conferences (C4) [21].

These tools improved accuracy, streamlined workflows, and enabled more timely referrals. Collectively, they supported the initiative’s shift toward a more structured, data-informed model of integrated care. Information sharing also matured during this period. TRiP employed a consent-driven approach that allowed partners to share client information internally, reducing duplication and improving coordination and efficiency:

“We’re able to share freely. It gives us background…and allows us to make sure we’ve got the right supports in place…When you’re working in silos, agencies are doubling up what they’re trying to do, and so you’re wasting resources…But when we work as TRiP, we work together to make sure we’ve got just what we need” [P11]

#### Persistent Challenges

Despite TRiP’s maturation and institutionalization during Phase 3, several structural and operational challenges persisted, constraining its ability to fully meet client needs. Persistent long wait lists limited timely access, often resulting in families disengaging before services became available. For clients unable to participate consistently, few alternative supports existed, leaving some youth without an appropriate point of referral.

Cross-sectoral alignment also remained a recurring difficulty. Partner agencies operated under different schedules, mandates, and capacity constraints, making coordinated planning challenging and leading to uneven engagement across organizations. This was particularly evident in declining participation in the Custom Coordinated Case Conferences (C4), where heavy workloads and limited staffing reduced some agencies’ ability to attend regularly.

High staff turnover and transience further complicated implementation, requiring continual rebuilding of relationships, re-establishing expectations, and maintaining trust within the partnership and with families:

“The challenge is there’s a lot of transiency in who is in the different roles, and that lack of consistency and continuity requires regular refreshing of those expectations, and also requires the continued rebuilding of relationships and trust.” [P1]

Information-sharing and privacy constraints presented additional challenges. Differences in institutional privacy rules and protracted approval processes for information-sharing agreements slowed both case coordination and evaluation activities. One participant explained:

“It is time-consuming. And anytime we have a project that requires data sharing, especially if it’s formal data sharing agreements and privacy impact assessments, it will have to be built into the timelines because it really slows things down. In some instances, there is no approval ever to link certain datasets.” [P3]

Finally, even with strengthened structures, youth exiting TRiP often experienced abrupt reductions in multi-agency support, exposing service gaps and highlighting broader system limitations in sustaining integrated care over time. These challenges collectively highlighted the importance of continuous adaptation, workforce continuity, and inter-organizational coordination in sustaining the initiative’s effectiveness and resilience.

### Phase 4: Deepening Integration, Innovation, and Resilience (2018–Present)

#### Deepening System Integration and Translating Learning into Practice

As TRiP matured, partners increasingly recognized that youth vulnerability required a broader ecosystem of support beyond the original health–education–justice partnership. This realization prompted the strategic inclusion of housing, addictions, recreation, child development, and parenting support services, resulting in a more holistic and family-oriented approach. Enhanced coordination enabled TRiP to address previously unmet needs, expand community-based programming, and compensate for staffing limitations through more distributed, cross-sector efforts. Over time, frontline experience generated a shared understanding of systemic gaps and inspired innovative solutions that had once seemed impossible. As one participant reflected, “*Working together has given us some new ideas on how we can collaborate to build supports for clients in ways we never thought of before*” [P20]. This accumulated knowledge increasingly informed managerial decision-making and contributed to broader conversations about reconfiguring service pathways for vulnerable youth and families.

Throughout this period, persistent challenges remained. Resource constraints and lengthy service waitlists sometimes limited timely access to services, requiring staff to prioritize high-risk youth and use flexible, distributed approaches to maintain client support. Even with strong collaborative structures, sustaining engagement across all partner agencies was an ongoing concern, as competing organizational priorities sometimes limited participation in joint problem-solving, highlighting the importance of champions and backbone staff to maintain momentum.

#### Strengthening Collaborative Structures and Accountability

By this phase, TRiP’s collaborative culture had become both more deliberate and more cohesive. Staff described a strong foundation of trust, open communication, and mutual respect that enabled difficult conversations and sustained joint problem-solving: “*We didn’t always agree, but we respected one another. And we had a great group*” [P5]. The initiative also refined its internal structures to maintain clear accountability and shared ownership. Regular communication was identified as a crucial enabler of collaboration. Regularly scheduled meetings also reinforced accountability. TRiP used four types of meetings for distinct purposes: weekly intersectoral Collaboration Team (iCT) meetings to identify resources and plan actions; biweekly Steering Committee meetings to monitor performance and plan long-term strategies; case conferences every four to six weeks to develop case management plans with clients; and weekly TRiP team (backbone team) meetings to coordinate upcoming activities. Participants emphasized that face-to-face engagement remained critical for holding one another accountable and ensuring follow through:

“The fact that they’re all sitting together, face to face, because when you’re exchanging emails, it’s easy to ignore, but when they work closely together, and they’re meeting literally every week, it’s a little harder to say, “No, that’s not me”, because they’re holding each other accountable that way.” [P8]

Throughout this period, the commitment of backbone staff and champions was essential to sustaining momentum and continuity during periods of organizational change. This commitment was particularly important to counteract lapses in engagement across agencies and ensure that collaborative problem-solving remained consistent despite competing organizational pressures.

#### Innovation, Adaptation, and Resilience

This phase also brought new efforts to expand preventive capacity and respond to service gaps. TRiP began developing a Hub Model of Collaborative Risk-Driven Intervention to provide rapid, short-term support for families who did not meet TRiP’s criteria but nevertheless faced acute risks. Designed as a city-wide preventative mechanism, the Hub represented a natural extension of TRiP’s shift toward a more integrated system of care and a more flexible model of early intervention. TRiP’s resilience was further tested during the COVID-19 pandemic, which temporarily slowed relationship-building, disrupted in-person meetings, and complicated the onboarding of new staff. Despite these setbacks, the initiative remained operational due to its established structures and strong relational foundation. Partners continued to collaborate in a “self-sustaining mode,” demonstrating the importance of stable governance, redundancy, and longstanding trust in maintaining collaborative work under crisis conditions.

Nevertheless, these innovations occurred in the context of ongoing challenges. Resource limitations and agency-level constraints meant that TRiP had to continuously adapt strategies to ensure service continuity. The initiative’s resilience was therefore not only a product of innovation but also of persistent problem-solving to navigate structural and organizational barriers.

## Discussion

This study provides a longitudinal analysis of the TRiP initiative, illustrating how a diverse set of human service organizations collaborated since 2010 to address the complex needs of vulnerable children and youth. Our findings highlight the dynamic interplay of structural, relational, and adaptive factors that enabled cross-sector collaboration, consistent with international evidence on integrated care. Specifically, our study extends the integrated care literature by examining how a multi-sectoral youth initiative evolved, adapted, and sustained over more than a decade. While prior research has identified key enablers of integrated care such as champions [35], shared goals, unifying visions, and common agendas [36], as well as flexibility, adaptive learning [37], and strong information-sharing and communication systems [38], much of this literature has focused on short-term implementation or single phases of integration. In contrast, this study demonstrates how these enabling factors interact dynamically over time across multiple developmental stages of collaboration.

The four phases of TRiP’s development map closely to Tuckman’s (1965) [14] model of group development (forming, storming, norming, and performing) while illustrating the overlapping and iterative nature of group dynamics and task-oriented actions. We distinguish between “group structure” (the relational and social dynamics among members) and “task-activity development” (organizational actions aimed at achieving initiative goals), recognizing that these dimensions interact continuously across time.

### Phase 1 and Early Forming

Phase 1 reflects the forming stage, during which partners collectively identified the problem of youth vulnerability and engaged in preliminary research to orient TRiP toward a shared vision. The coalescence around the goal of improving the safety and well-being of children and youth provided the foundation for early collaboration. The literature on integrated care emphasizes the importance of leadership and boundary-spanning actors [39], and our findings similarly demonstrate how champions played a critical role in initiating the partnership, navigating diverse institutional norms, mobilizing partners, and facilitating open dialogues across sectors. However, despite strong leadership, organizational silos and resource limitations persisted as important barriers during the early stages of collaboration. Equally central to this phase were the alignment of values and early trust-building, which enabled TRiP to establish foundational governance structures, including the first Steering Committee, setting the stage for structured collaboration and coordinated action.

### Phase 2 and Early Implementation

In Phase 2, the initiative transitioned from planning to implementation, marking movement toward the performing stage in task-activity development. The iterative refinement of operational tools and processes enabled TRiP to adjust service delivery in real time. Improvements in reporting, monitoring, and co-location of partners strengthened both information flow and relational trust, reflecting the norming stage of group development. Our findings build on existing research highlighting the importance of flexibility in sustaining long-term integrated care initiatives [40] by demonstrating how adaptive learning and responsiveness supported TRiP’s growth during Phase 2.

As implementation progressed, previously unrecognized service gaps and client needs became more visible, requiring the initiative to adapt and incorporate new responses. This occurred through program expansion, including the extension of services to adolescents through the twelve&up program. Importantly, flexibility operated alongside increasing organizational formalization and governance development. TRiP combined adaptive approaches with structural consolidation, including the introduction of a coordinator role. This balance between flexibility and formalization enabled the initiative to evolve while maintaining organizational stability.

### Phase 3 and Evaluation-Driven Refinement

Phase 3 exemplifies the interplay of storming and norming stages, driven by formal evaluation and internal reflection. The literature on cross-sector partnerships [41,42] highlights the ongoing tensions in collaborative networks, presenting “storming” dynamics as recurring rather than one-time events. Based on our findings, storming dynamics are present throughout various phases, including phase 3. Despite structural improvements through comprehensive mixed-methods evaluation which facilitated accountability, highlighted gaps, and prompted targeted adaptations (such as the Student Engagement Worker role), challenges persisted. These include uneven partner engagement, high staff turnover, and complex privacy constraints. The recurrence of challenges should not be viewed as a failure of collaborative development. Rather, the findings suggest that collaborative integrated care initiatives move through cyclical and overlapping developmental processes, challenging strictly linear interpretations of Tuckman’s model.

Through evaluation, TRiP also entered the performing stage in task-activity development, implementing refined procedures and expanded services that leveraged the knowledge and relationships developed in earlier phases. This highlights the importance of evaluation not only as a measurement tool but as a mechanism to institutionalize learning and strengthen collaboration [43]. Our findings further suggest that regular evaluation functions as an important mechanism that can respond to the “storming” dynamics in a generative manner.

### Phase 4 and Sustained Performing

Phase 4 reflects an advanced performing stage, marked by deeper system integration, service diversification, and collaborative resilience. During this phase, TRiP broadened its ecosystem to include housing, addictions, recreation, and parenting supports, signaling a transition toward a more holistic, family-oriented model of integrated care. This evolution, alongside the earlier introduction of the Student Engagement Worker role, illustrates how collaborative structures adapt by incorporating new partners and positions to address unmet needs and sustain coordinated action, an approach consistent with recent scholarship on agile, network-based models of integrated care [40,41,42]. In the case of TRiP, this adaptive capacity was strengthened by years of relationship-building and accumulated collaborative experience, processes that may require substantial time and sustained investment in other integrated care initiatives.

The development of preventative mechanisms, such as the Hub Model, further demonstrates TRiP’s maturing capacity to anticipate risk and mobilize cross-sector resources proactively. The initiative also showed notable resilience during system shocks, including the COVID-19 pandemic, aligning with emerging evidence that long-standing interorganizational networks with strong trust and communication channels are better positioned to adapt under crisis conditions [43].

Despite its maturation, TRiP continued to encounter persistent challenges, including resource constraints, uneven partner participation, and staff turnover. In long-running collaborations, turnover disrupts relational continuity and often necessitates repeated cycles of trust-building and role clarification. As such, performing is not a stable end state but an iterative process in which group structure and task-activity development must continuously realign. Our findings suggest that formal accountability processes, sustained communication channels, and intentional relationship maintenance remain essential to mitigating vulnerability to organizational and system-level disruptions and ensuring continuity in integrated care delivery [40,41,42,43,44].

## Limitations

Several limitations of this study should be noted. **First**, this study employed a single-case study design focused on one collaborative initiative (TRiP) in Regina, Saskatchewan. While the case provides rich insights, findings may not be generalizable to other jurisdictions with different institutional arrangements, policy environments, or community contexts. **Second**, the findings primarily reflect the perspectives of policymakers and service providers. Although triangulated through document reviews and observations, the absence of direct accounts from children, youth, and families limits the representation of users’ lived experiences in this paper. These perspectives are examined separately in another publication dedicated to TRiP users. **Third**, as interviews were conducted primarily during 2023, the findings reflect a particular moment in TRiP’s evolution; future changes in funding, leadership, or policy could alter the initiative’s dynamics. **Finally**, while thematic analysis provided rich insights into cross-sectoral collaboration, the study was not designed to assess quantitative outcomes. This limits the ability to evaluate TRiP’s long-term effectiveness or directly compare it with other integrated care models. Future mixed-methods and longitudinal research could help address this gap.

## Conclusion and Implications for Integrated Care

TRiP’s evolution demonstrates that integrated care is not a linear or static process, but an ongoing and adaptive form of collaboration shaped by changing needs, organizational learning, and recurring challenges. Mapping TRiP’s development onto Tuckman’s model illustrates how forming, storming, norming, and performing phases frequently overlap and re-emerge as partnerships evolve, new members join, and service demands shift. TRiP offers an important example of how integrated youth service collaborations can be institutionalized and sustained over time within a fragmented service environment while maintaining responsiveness to vulnerable children, youth, and families.

Our findings highlight the critical role of champions, shared vision, and relational trust in establishing and sustaining cross-sector collaboration. Champions were instrumental in mobilizing partners, navigating institutional boundaries, facilitating difficult conversations, and maintaining momentum during periods of uncertainty and organizational change. However, TRiP’s longevity also depended on moving beyond reliance on individual leaders toward more formalized governance structures, accountability mechanisms, and organizational supports capable of sustaining collaboration despite staff turnover and shifting institutional priorities. These findings reinforce the importance of combining relational leadership with stable structural infrastructure in long-term integrated care initiatives.

The study further demonstrates that adaptive learning, evaluation, and information-sharing systems are central to sustaining integrated care over time. In TRiP’s case, evaluation functioned not only as a measurement tool but also as a mechanism for organizational learning, refinement, and resilience. Continuous reflection enabled the initiative to identify emerging gaps, adapt services, strengthen coordination, and institutionalize collaborative practices. Co-location, regular communication, and structured accountability processes also supported relational continuity and timely problem-solving, while flexible governance enabled the initiative to respond to evolving community needs and system shocks, including the COVID-19 pandemic.

Overall, TRiP contributes longitudinal empirical evidence to the growing international literature on integrated care by demonstrating that sustainable collaboration requires both organizational formalization and relational flexibility. The initiative highlights the importance of balancing shared vision with adaptability to emerging vulnerabilities and changing community needs.

These findings offer practical lessons for jurisdictions seeking to move beyond fragmented youth mental health and social care systems toward more coordinated, prevention-oriented, and relationship-based models of integrated care.

## Data Availability

The data that has been used is confidential.
